# Quantifying personal exposure to traffic and household air pollution: a pilot study among street traders in Lagos, Nigeria

**DOI:** 10.7189/jogh.16.04114

**Published:** 2026-04-17

**Authors:** Obianuju B Ozoh, Prince Amegbor, Sandra K Dede, Olufunke O Adeyeye, Ogochukwu A Ekete, Olorunfemi Adetona, Benjamin Barrat

**Affiliations:** 1 Department of Medicine, College of Medicine, University of Lagos, Lagos, Nigeria; 2 Department of Medicine, Lagos University Teaching Hospital, Idi-Araba, Lagos, Nigeria; 3 Department of Global and Environmental Health, School of Global Public Health, New York University, New York, USA; 4 Department of Population Health Sciences, Kings College, London, UK; 5 Department of Medicine, Lagos State University College of Medicine, Ikeja, Lagos, Nigeria; 6 Department of Medicine, Lagos State University Teaching Hospital, Ikeja, Lagos, Nigeria; 7 Division of Environmental Health Sciences, College of Public Health, The Ohio State University, Columbus, Ohio, USA; 8 Environmental Research Group, School of Public Health, Imperial College London, London, UK

## Abstract

**Background:**

Ambient and household air pollution (HAP) exposure is associated with adverse health outcomes. Heavy traffic congestion, street trading, and the use of biomass fuels are common in Lagos, Nigeria. We designed this study to evaluate the feasibility of measuring prolonged personal exposure to air pollution among street traders. We aimed to quantify average 24-hour exposure to air pollution and evaluate how proximity to major highways influences these levels.

**Methods:**

We conducted a cross-sectional study assessing recruitment and level of personal exposure to traffic-related air pollution (TRAP) and HAP among adult street traders over 48 hours. We recruited ‘highway traders’ (<50 m of the highway) and ‘inner street traders’ (>200 m from the highway) and measured personal exposure to particulate matter (PM_2.5_ and PM_10_) using a bespoke monitoring unit held in a backpack. We also measured blood pressure (BP) and performed spirometry.

**Results:**

The recruitment rate was 33%, yielding a sample of 30 females (15 per group). All eligible males declined due to time constraints and because they found the backpack monitor unacceptable. The average 24-hour PM_2.5_ (mean (x̄) = 34.3 μg/m^3^) and PM_10_ (x̄ = 34.7 μg/m^3^) exposures for highway traders were higher than PM_2.5_ (x̄ = 22.8 μg/m^3^) and PM_10_ (x̄ = 23.0 μg/m^3^) exposures for inner street traders. The PM_2.5_ over 24 hours was consistently above the World Health Organization recommended threshold of 15ug/m^3^ for both groups with diurnal peaks. Spirometry parameters and BP were similar between the two groups.

**Conclusions:**

Device acceptability and measurement site selection are pivotal in participant recruitment. Successful gender-inclusive recruitment necessitates appropriate monitoring equipment and measurement at sites convenient for participants. All street traders experienced consistently high air pollution levels throughout the day, regardless of proximity to the highway. These findings provide valuable insights to guide future large-scale research.

The World Health Organization (WHO) reports that 99% of people worldwide are exposed to air pollution levels above recommended thresholds [1]. In 2019, exposure to both ambient air pollution (AAP) and household air pollution (HAP) contributed to 6.7 million deaths worldwide [1]. The burden of exposure and adverse effects is unevenly distributed, with 89% of deaths occurring in low and middle-income countries [2]. Specifically, in Africa, there were 1.1 million estimated deaths due to air pollution in 2019 [3]. Rapid urbanisation, poverty, and other adverse social determinants of health in Africa worsen these effects [4]. Fine particulate matter (PM) with an aerodynamic diameter <2.5 μm (PM_2.5_) is an important component of traffic-related air pollution (TRAP) and has been linked to the onset and progression of respiratory diseases, cardiovascular, metabolic, and neurological diseases [5]. It can penetrate deep into the lungs and translocate into the bloodstream, where it increases oxidative stress, alters immune function, and induces epigenetic changes [6,7]. The adverse health effects of TRAP are most pronounced within 200 m from a highway or major road in the upwind direction and within 300–500 m in the downwind direction [8].

Lagos is a major Nigerian city and ranks among the fastest-growing cities worldwide, with its population projected to reach 32 million by 2050 [9]. The city is cosmopolitan, densely populated, and has persistent traffic congestion. About 227 vehicles/km/d ply Lagos roads, as road transportation remains the primary mode of transport [10]. In addition, most of the vehicles are imported, used, and old cars, trucks, and motorcycles that often use high-sulfur fuels [1]. It is estimated that TRAP contributes 30% and HAP 19% of AAP in Lagos [10]. Low-income households constitute about two-thirds of the Lagos population, and HAP is attributed to their continued reliance on biomass as primary cooking fuel [11,12].

In Lagos, the informal sector accounts for about 65% of the working population, with many individuals living or working close to highways or major roads [13]. Street trading is a prevalent occupation that requires individuals to spend long hours daily near congested highways. This proximity places them at high risk of intense exposure to TRAP and its associated adverse health outcomes [14,15]. Evidence suggests that indoor exposure levels are generally lower than outdoor levels [16], but there is limited understanding of TRAP and HAP exposure levels among street traders in Lagos. The scarcity of data impedes the development and implementation of effective protective policies for this vulnerable population.

We aimed to evaluate the feasibility of conducting a future large-scale study in Lagos to assess exposure to and the effects of TRAP. We assessed the feasibility of participant recruitment across genders, adherence to prolonged personal air pollution monitoring, and collection of clinical data. We also quantified personal exposure to PM_2.5_ based on proximity to the highway and categorised it by approximate time spent at work and at home, as a surrogate for comparing exposure to AAP from TRAP and HAP.

## METHODS

### Study design and setting

We conducted a cross-sectional study to assess street traders’ recruitment and personal exposure to air pollution over 48 hours.

**Figure 1:**
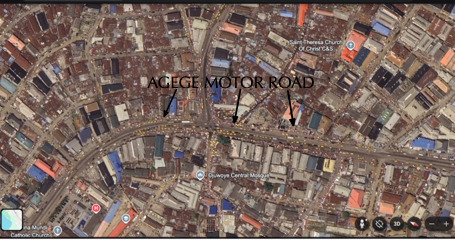
Aerial view of Mushin market. Source: Sandra K Dede, with permission.

**Figure 2:**
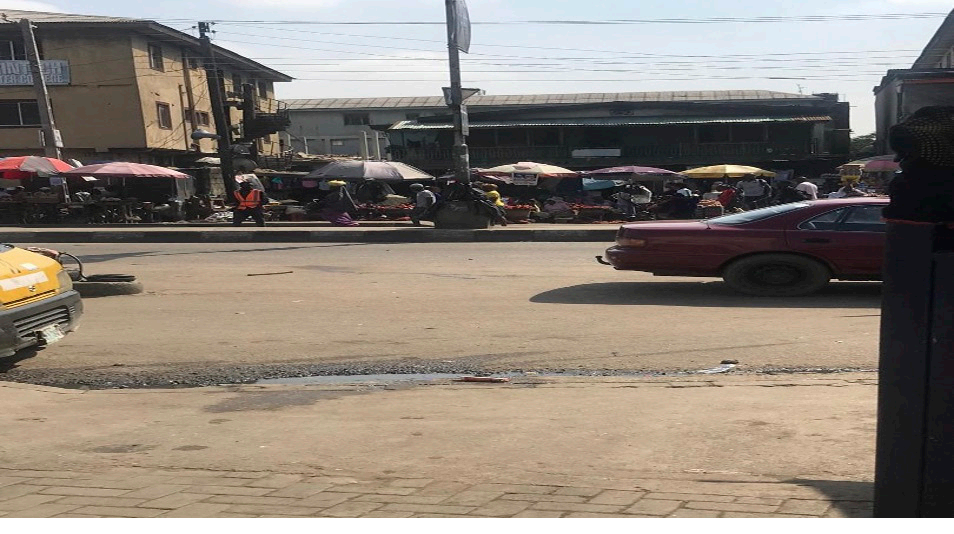
Roadside trading in Mushin market, Lagos, Nigeria, showing traders directly beside the highway under umbrellas. Source: Obianuju B Ozoh, with permission.

The Mushin axis of the Agege motor road is a recognised high traffic hub in Lagos (**Figure 1**). It is plied by heavy diesel trucks, cars, commercial buses, and motorbikes. It crosses a very busy market where street trading takes place directly on the roadside, with no structural barrier separating it from the road (**Figure 2**). The highway street traders comprise both men and women, and they do not use any personal protective equipment to reduce exposure to TRAP. In the inner streets of Mushin, street trading also takes place along less-trafficked residential streets, with most traders living in the houses behind their stores.

### Participants

We divided street traders into two groups: ‘highway traders’ (<50 m from the highway) and ‘inner street traders’ (>200 m away from the highway).

Male and female traders aged ≥18 years were eligible to participate. We included non-tobacco smokers and those who had worked as a street trader for at least one year and excluded pregnant women and those with reported exposure to another dusty occupation, such as street sweeping or construction work. We recruited consecutive traders who met the inclusion criteria and consented to complete all aspects of the study. All participants received monetary compensation

As this was a pilot study, we recruited 30 participants (15 per group) to assess the feasibility of conducting a larger-scale study.

### Data collection

We collected the data between 13 and 29 October 2021. We trained the field workers to administer the questionnaire, set up the air pollution monitoring device, and measure height, weight, and blood pressure (BP) in the field. We subsequently invited the participants to the Lagos University Teaching Hospital, located within 500 m of the study site, for spirometry testing within one week of the air pollution monitoring.

### Questionnaire administration

We used a structured questionnaire to collect the sociodemographic information, including level of education, duration of street trading, and hours spent at work. We used the International Multidisciplinary Programme to Address Lung Health and TB in Africa respiratory symptom and life exposure questionnaires to assess respiratory symptoms (*i.e.* occurrence, type, and frequency of respiratory symptoms in the previous year) and exposure to air pollution from cooking fuel and daily living exposures. The African lung health researchers developed the questionnaire, and it has been found reliable for assessing respiratory symptoms and exposures among Africans [17].

### Measurements

#### Personal air pollution exposure monitoring

We measured personal exposure using a bespoke backpack (Dyson, Malmesbury, UK) that incorporates a small air pollution monitoring unit with an inbuilt global positioning system data logger (Figure S1 in the **Online Supplementary Document**). A large-scale study has previously used it among school children across six African countries [18], but the feasibility of use among adults had not been assessed.

We trained the participants to safely carry the backpack for 48 hours, both at home and at work, and while commuting, and to place it beside them while sleeping or bathing. We considered the 48-hour monitoring duration adequate because it is long enough to capture day-to-day variations in activity and microenvironments, in addition to being logistically more feasible compared to longer durations of monitoring [19].

The bespoke backpack measures PM_10_, PM_2.5_, and nitrogen dioxide using a monitor placed in the front pocket (Figure S1 in the **Online Supplementary Document**). It logs and stores pollutants at a one-second interval. We calibrated all backpack monitors by co-location with a reference monitor for four days in London, UK, before use [18].

We used six devices and conducted personal exposure monitoring for 48 hours for each participant. We simultaneously conducted the monitoring (three highway traders and three inner-street traders) to control for meteorological variables that could influence air pollution levels. We only used the data for exposure to PM_2.5_ and PM_10,_ as high uncertainty was previously reported for nitrogen dioxide measures during calibration [18].

#### Anthropometry and BP measurement

We measured height, weight, body mass index, and BP following the WHO stepwise approach to chronic disease surveillance protocol [20]. We measured BP using Omron® sphygmomanometers calibrated before first use and at the beginning of every week thereafter. We recorded BP three times (1–3 minutes apart), documenting only the average of the last two readings. We defined hypertension as systolic/diastolic measurements ≥140/90 mm Hg or a previous physician diagnosis.

#### Spirometry testing

Following the ATS/ERS guidance, trained and certified personnel did the pre- and post-bronchodilator spirometry using a vitalograph® pneumotrac spirometer [21]. We calibrated the spirometer daily and individually reviewed all tests for quality assurance, including only those that met the acceptability criteria. We recorded the forced expiratory volume in one second (FEV1), forced vital capacity (FVC), the FEV1/FVC ratio, and the forced expiratory flow at 25–75% of FVC. We also tested pre- and post-bronchodilator (after 400 ug of salbutamol).

### Data analysis

We conducted descriptive statistics for sociodemographic and clinical characteristics and PM exposure among all participants, comparing them by trader type using the Mann-Whitney test and χ^2^ test as appropriate. Specifically, we calculated the mean (x̄) and median (MD) 24-hour exposure values. Additionally, we conducted descriptive analyses to show exposure levels by location, examining x̄ and MD exposure values for each group during working hours (8 am to 7 pm) and outside these hours (considered as home exposure). We also plotted exposure over the study period against the maximum WHO 24-hour recommended exposure level of 15ug/m^3^ [22].

We used *R*, version 4.4.1 (R Core Team, Vienna, Austria) for all analyses.

## RESULTS

Of the 90 traders approached (30 of whom were men), we successfully recruited 30 participants. This represents a 33% overall participation rate, with 50% among women and none among men. Only female traders consented to participate in the study; all male traders declined, citing time constraints (particularly the need to travel to the hospital for spirometry) and the awkwardness of carrying a backpack.

All 30 participants successfully completed anthropometry measurement and 48 hours of personal air pollution exposure monitoring. All 48-hour records were complete, with no missing data at home or at work. The backpacks were fully charged for 12–16 hours after each participant's use, to ensure reliability. There was no record of device malfunction over the study duration. A total of 27 participants completed spirometry, of which one declined a post-bronchodilator test. Three highway traders declined spirometry due to time constraints.

The x̄ age of participants was 42.8 (standard deviation = 10.0), and the MD was 44 (interquartile range = 11.5) years (**Table 1**). Most (93.3%) participants were married, and over half (53.3%) had attained secondary education. The MD monthly income was NGN 27 500, translating to approximately USD 69.1 based on the October 2021 exchange rate of USD 1 = NGN 398.

**Table 1:** Summary of study variables by type of traders*

	Inner street traders	Highway traders	*P*-value†
**Ambient PM exposure in μg/m^3^**			
PM_2.5_, x̄ (SD)	22.8 (6.2)	34.3 (21.9)	0.069
PM_2.5_, MD (IQR)	17.9 (11.0–22.2)	18.3 (12.8–23.6)	0.512
PM_10_, x̄ (SD)	23.0 (6.4)	34.7 (22.3)	0.070
PM_10_, MD (IQR)	17.9 (11.0–22.2)	18.3 (12.8–23.6)	0.534
**Socioeconomic characteristics**			
Age in years, x̄ (SD)	40.5 (9.1)	45.1 (10.6)	0.220
Marital status			
*Married*	14 (93.3)	14 (93.3)	1.000
*Single*	1 (6.7)	1 (6.7)	
Highest level of education			
*None*	0 (0)	2 (13.3)	
*Primary*	0 (0)	1 (6.7)	
*Some secondary*	2 (13.3)	2 (13.3)	0.458
*Completed secondary*	8 (53.3)	8 (53.3)	
*Technical post-secondary*	4 (26.7)	2 (13.3)	
*Postgraduate degree*	1 (6.7)	0 (0)	
Monthly income in NGN, x̄ (SD)	45 533.30 (28 811.9)	23 133.30 (15 282.4)	0.024
**Respiratory symptoms**			
Cough on most days			
*Yes*	0 (0.0)	0 (0.0)	
*No*	15 (100.0)	15 (100)	
Phlegm on most days			
*NA*	12 (80.0)	13 (86.7)	
*Yes*	0 (0.0)	1 (6.7)	0.361
*No*	3 (20.0)	1 (6.7)	
Wheeze in the past 12 months			
*Yes*	0 (0)	2 (13.3)	0.464
*No*	15 (100.0)	13 (86.7)	
**Air pollutants exposures**			
Main cook			
*Yes*	11 (73.3)	11 (73.3)	1.000
*No*	4 (26.7)	4 (26.7)	
Main cooking fuel			
*Wood*	1 (6.7)	0 (0.0)	
*Kerosene*	2 (13.3)	0 (0.0)	0.189
*Gas*	12 (80.0)	15 (100.0)	
Exposure to vapour, dust, and fumes >15 hours per week			
*Yes*	5 (33.3)	15 (100.0)	<0.001
*No*	10 (66.7)	0 (0.0)	
Exposure to smoke (burning waste)			
*Yes*	2 (13.3)	15 (100.0)	<0.001
*No*	13 (86.7)	0 (0.0)	
Exposure to aerosols			
*Yes*	13 (86.7)	10 (66.7)	0.357
*No*	2 (13.3)	5 (33.3)	
Exposure to fumes (mosquito coils)			
*Yes*	4 (26.7)	10 (66.7)	0.067
*No*	11 (73.3)	5 (33.3)	
Generator within 50 m of sleeping or living area			
*Yes*	11 (73.3)	8 (53.3)	0.449
*No*	4 (26.7)	7 (46.7)	
**Health outcomes**			
Diagnosed with hypertension			
*Yes*	6 (40.0)	2 (13.3)	0.215
*No*	9 (60.0)	13 (86.7)	
Hypertension status (BP reading)			
*Yes*	7 (46.7)	6 (40.0)	1.000
*No*	8 (53.3)	9 (60.0)	
Systolic BP, x̄ (SD)	147.9 (38.8)	132.4 (132.4)	0.172
Diastolic BP, x̄ (SD)	90.2 (21.5)	84.5 (10.1)	0.362
BMI, x̄ (SD)	26.3 (6.6)	29.0 (8.5)	0.362
Pre-FEV1% predicted, x̄ (SD)	81 (19.7)	76.2 (15.5)	0.489
Post-FEV1% predicted, x̄ (SD)	83.5 (19.9)	82 (14.4)	0.829
Pre-FVC % predicted, x̄ (SD)	83.7 (18.3)	78.5 (13.9)	0.413
Post-FVC % predicted, x̄ (SD)	85.6 (18.3)	80.5 (12.9)	0.417
Pre-FEV1/FVC % predicted, x̄ (SD)	94.5 (7.1)	93.4 (9.3)	0.734
Post-FEV1/FVC % predicted, x̄ (SD)	97.7 (9.09)	98.3 (8.44)	0.878
Pre FEF % predicted, x̄ (SD)	75.3 (30.7)	69.6 (31.9)	0.644
Post FEF % predicted, x̄ (SD)	80.4 (35.4)	95.5 (33.5)	0.281

BMI –body mass index, BP – blood pressure, FEF – forced expiratory flow, FEV1 – forced expiratory volume in one second, FVC – forced vital capacity, IQR – interquartile range, MD – median, PM – particulate matter, SD – standard deviation, x̄ – mean

*Presented as n (%) unless specified otherwise.

†*P*-values are from a χ^2^ test for categorical and a Wilcoxon test for numerical variables

The average 24-hour PM_2.5_ (x̄ = 34.3 μg/m^3^) and PM_10_ (x̄ = 34.7 μg/m^3^) exposures for highway traders were higher than PM_2.5_ (x̄ = 22.8 μg/m^3^) and PM_10_ (x̄ = 23.0 μg/m^3^) exposures for inner street traders, but the difference was not statistically significant. However, MD exposure for PM_2.5_ and PM_10_ was similar in both groups. Furthermore, the average monthly income (in NGN) was higher for inner street traders (MD = 27 500) than for highway traders (MD = 17 500). Additionally, all inner street traders had completed secondary education or higher. According to BP readings, 46.7% of inner street traders and 40% of highway traders had hypertension, while 40% of inner street traders and 13% of highway traders reported a previous diagnosis. All spirometry parameters were similar between the groups, with no significant differences in percentage-predicted values.

Participants reported an average work duration of 11 hours (8 am to 7 pm). The MD PM concentration at home was significantly higher among inner street traders than among highway traders (**Table 2**). However, highway traders had higher MD PM_10_ concentrations at home and at work than inner street traders. Specifically, highway traders had higher average PM_2.5_ concentrations at home (MD = 41.18 μg/m^3^) and at work ( MD = 29.20 μg/m^3^), compared to the PM2.5 concentrations at home (MD = 27.79 μg/m^3^) and at work (MD = 18.78 μg/m^3^) of inner street traders.

**Table 2:** Summary statistics of exposure by location across groups*

	Home	Work	*P*-value†
**PM_2.5_**			
Inner street	21.5 (12.8–33.6)	12.4 (7.4–22.0)	0.000
Highway	17.1 (3.8–32.4)	20.0 (12.6–31.9)	0.454
**PM_10_**			
Inner street	21.6 (12.8–33.7)	12.5 (7.4–22.4)	0.000
Highway	17.2 (3.9–32.4)	20.0 (12.6–32.0)	0.460

IQR – interquartile range, MD – median, PM – particulate matter

*Presented as MD (IQR).

†*P*-values are from paired Wilcoxon signed-rank tests comparing home and work exposures within participants, stratified by trader type.

**Figure 3:**
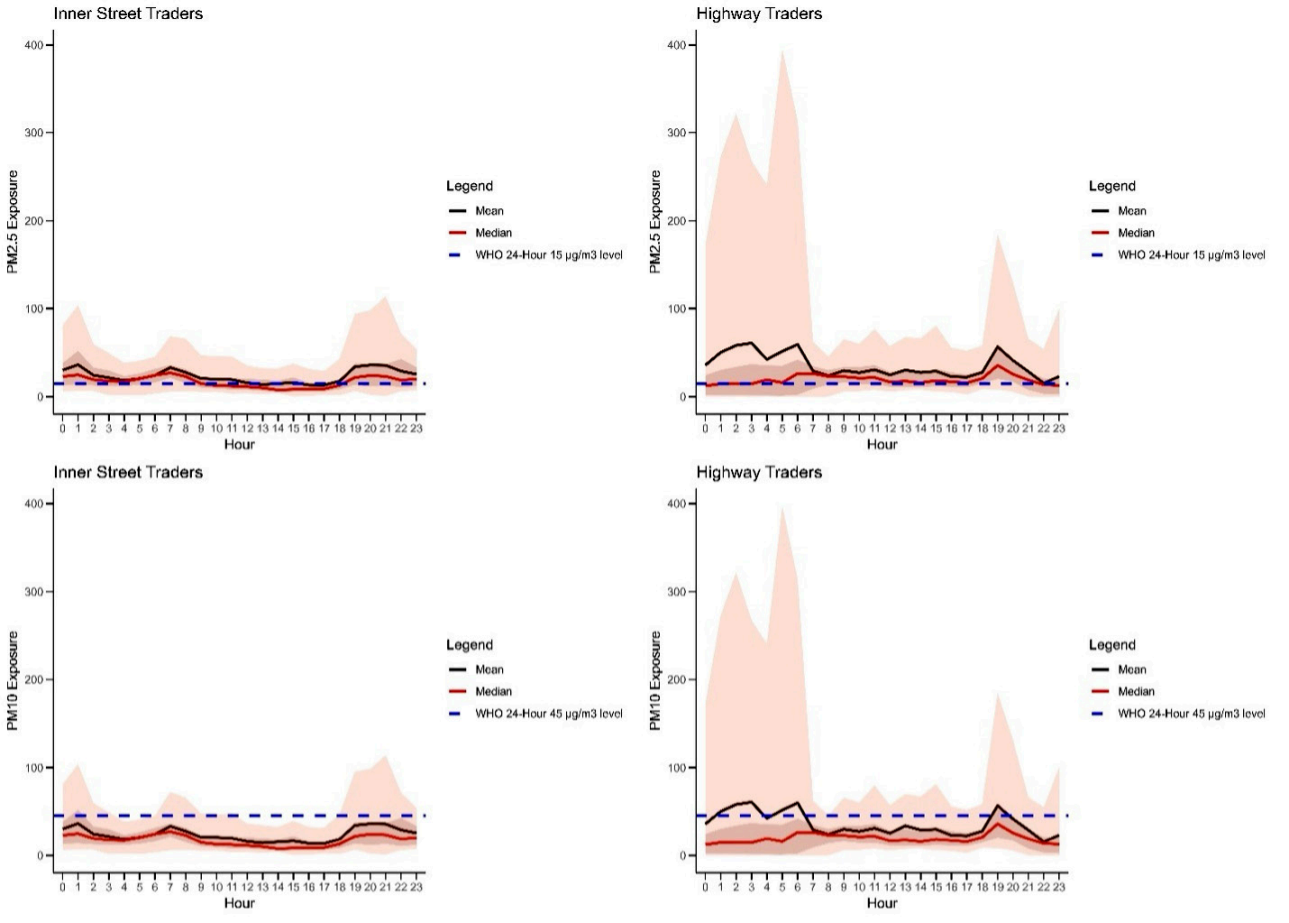
Diurnal patterns of hourly PM_2.5_ and PM_10_ exposure for highway and inner street traders over a 24-hour period. **Panel A.** PM_2.5_ exposure for inner street traders. **Panel B.** PM_2.5_ exposure for highway traders. **Panel C.** PM_10_ exposure for inner street traders. **Panel D.** PM_10_ exposure for highway traders. The light coral shade represents the 95% confidence interval for exposure values, while the darker shade indicates the interquartile range. PM – particulate matter.

The average (x̄) exposure to PM_2.5_ exceeded the WHO threshold for most of the 24 hours, with diurnal spikes in the morning and evening for both groups (**Figure 3**, Panels A and B). Inner street traders had lower peak levels over the 24 hours. The x̄ PM_10_ had diurnal spikes above the WHO threshold only among highway traders with higher peak levels over the 24 hours (**Figure 3**, Panels C and D).

## DISCUSSION

We conducted one of the few studies to quantify prolonged AAP exposure among these occupationally exposed vulnerable individuals in West Africa. Regarding feasibility, the recruitment rate was low with a wide gender disparity. All eligible men declined to participate because carrying a backpack and leaving their businesses for hospital-based measurement were unacceptable. The x̄ daily level of exposure to PM_2.5_ for all participants (highway and inner street traders) exceeded the WHO-recommended 24-hour average threshold of 15 μg/m^3^, with pronounced morning and evening spikes that were higher among the highway traders. The x̄ PM_10_ also showed diurnal spikes that were above the WHO threshold only among highway traders.

We uncovered several important aspects related to the feasibility of participant recruitment and the acceptability of personal air pollution monitors. Generally, the participation rate was low, and the gendered recruitment barrier was high, with all men declining to participate. Although women also found wearing the backpack burdensome, they were more inclined to accept it. The experience corroborates the findings from a systematic review that factors such as monitor size and portability significantly impact acceptability [23]. The study reported that ‘the size of wearable devices and how they are carried were two of the most important factors influencing users’ acceptance of wearable sensors’ [23]. Another study noted that potential study participants want wearable devices that are small, worn like a watch, and no larger than the palm, with some preferring them when worn as jewellery [24]. Therefore, the current form of the backpack device we used may be inappropriate for future large-scale studies involving adults. Instead, a more discreet and lighter device will be necessary to improve participation. Interestingly, our findings regarding acceptability differ from those of an earlier study involving schoolchildren across six African countries, in which both boys and girls readily accepted the same backpack monitor [18]. For children, the similarity to a typical school bag likely contributed to acceptability.

Another consideration in air pollution monitoring device selection is the reliability and accuracy of sensors in capturing pollutant levels, along with the ease of accessing recorded data [23]. The customised backpack device we used proved reliable, efficient, and practical, particularly in low-resource settings, because remote data access was possible. Hence, the most crucial modification required for the bespoke backpack is the packaging of the sensor into a more portable device.

An additional insight we gained regarding feasibility is that research procedures requiring participants to leave their businesses for extended periods decrease participation rates. Notably, two-thirds of eligible individuals declined to participate because hospital-based spirometry testing required them to be away from their businesses for a few hours. Time constraints emerged as a predominant reason for this non-participation, with men more frequently citing a lack of time than women. This may be attributed to the significant financial responsibilities placed on men and the high levels of poverty, which necessitate prioritising income-generating activities over research participation [25]. Culturally, many African men also tend to avoid hospital visits due to concerns about stigma [26]. While community-based spirometry testing is feasible and has been successfully employed in previous large-scale studies [27], conducting spirometry directly along the highway may be logistically challenging. It is apparent that, for future large-scale research, it will be important to locate all study procedures, including spirometry, closer to participants’ workplaces or residences to enhance participation. The use of mobile clinics has demonstrated effectiveness in improving recruitment and participation in other research settings [28], and we anticipate using this approach to enhance participation in future studies.

Personal exposure monitoring is a more accurate assessment of air pollution exposure than fixed-site monitoring, as it accounts for variables such as gender, daily routines, individual behaviours, and locations [2]. This approach is particularly valuable for cohort and panel studies, where detailed health data are collected at the individual level [3]. By capturing these nuances, personal monitoring deepens understanding of the health impacts of air pollution. Most studies among street traders in Africa have used fixed monitors to estimate exposure to air pollution. A study in Ghana used fixed monitors along traffic corridors to measure AAP levels and found a higher risk of respiratory and cardiovascular disease among traders than among office workers [4]. However, the confounding effects of differences in overall daily exposure, including HAP exposure, as well as disparities in other social determinants of health between the two groups, were not considered.

We found that average daily exposure to PM_2.5_ for both highway and inner street traders consistently exceeded the WHO-recommended 24-hour threshold of 15 ug/m^3^. The raised exposure occurred both during the expected time at work and at home. While highway traders experienced somewhat higher average exposures than their inner street counterparts, especially during peak traffic periods, all traders faced potentially harmful conditions. This underscores that street trading is a potentially risky occupation, regardless of traders' proximity to major highways. Although inner street traders may experience less peak traffic exposure, they are still affected by residual highway pollution, influenced by meteorological factors such as wind speed and direction, and by local street traffic. Additionally, many inner street traders reside at the same locations as their shops, resulting in ongoing exposure to TRAP outside of work hours. It is important to recognise that other factors may contribute to air pollution exposure among street traders in Lagos. These include the home use of biomass fuels for cooking and electricity generation, and raise the question of whether HAP exposure can exceed or closely mirror TRAP encountered through street trading.

A study in urban Johannesburg found that outdoor food vendors experienced higher air pollution exposure compared to their indoor counterparts, suggesting that AAP is a major contributor to overall exposure [29]. Furthermore, the CLEAN-Air study in Cameroon highlighted the significant contribution of TRAP to the level of personal air pollution exposure [30]. It showed that residing near major roads can mask the differences in exposure levels between individuals using clean cooking fuels and those using more polluting options, underscoring the need for a comprehensive strategy to reduce both AAP and HAP in urban African cities [30]. To effectively reduce TRAP, there is a need for improved traffic management and regulations to limit the use of old, highly emitting vehicles. Additionally, the government needs to invest in alternative public transportation systems that use cleaner energy and adopt modern urban planning methods that avoid siting markets, schools, and residential buildings along busy highways. Moreover, improving electricity supply, expanding access to clean cooking energy and enforcement of building standards that encourage cross ventilation, particularly in low-resource areas, are all strategies that can reduce HAP and AAP [31]. Community education about the adverse health effects of air pollution is also essential and will promote citizen science and empower individuals to participate in collective and individual actions to lower their personal exposure risk.

We found no significant differences in spirometry parameters between the two groups of traders, although highway traders exhibited pre-bronchodilator FEV1 and FVC values <80% of predicted, suggesting a possible restrictive impairment. Restrictive spirometry or small lungs is a recognised key determinant of cardiometabolic and all-cause mortality, and future research should incorporate assessment of the cardio-metabolic effects of TRAP [32,33].

This study has several limitations. First, the absence of a time activity diary precluded more precise documentation of time spent at work, at home, and during commutes, thereby hindering accurate clarification of the contributions of different pollution sources across different microenvironments. We relied on an estimated work period from 8 am to 7 pm, based on the average hours reported by participants, acknowledging that schedules varied. Commuting is also likely to contribute more to TRAP exposure among ‘highway traders’ who may travel longer distances to the markets compared to their ‘inner street’ counterparts who usually reside close to their stalls. Nevertheless, by analysing average 24-hour PM exposure, our findings indicate that participants were exposed to potentially harmful levels of PM_2.5_ throughout most of the day. Second, the small sample size makes the reported exposure data and associations exploratory. Additionally, we acknowledge that seasonal changes in traffic intensity, congestion patterns, and meteorological conditions could modify the magnitude of observed exposure gradients. Our findings should therefore be interpreted as representative of short-term conditions during the sampling period rather than year-round averages. Also, multi-day personal monitoring (up to seven days) could reduce the influence of day-specific behavioural patterns, such as cooking activities and generator use, and better characterise typical exposure. However, we were unable to extend monitoring beyond 48 hours due to logistical challenges, but this should be considered in future studies. Despite these limitations, we provide valuable insights into recruitment strategies and the study approach to inform planning for future well-powered studies.

## CONCLUSIONS

We highlight important considerations for future research on personal exposure to air pollution among street traders. Achieving adequate participant recruitment across genders requires not only the use of monitoring devices that are acceptable and comfortable for all participants but also conducting all measurements in locations convenient to them. The backpack we used may present acceptability barriers, especially among men. Regarding air pollution exposure, ‘highway traders’ tended to experience higher exposure during periods of heavy traffic compared to ‘inner streets traders’. However, it is noteworthy that all street traders, regardless of location, were exposed to air pollution levels exceeding daily recommended thresholds, with HAP contributing substantially.

## Additional material


Online Supplementary Document
