# Supplementary material for: Quantifying personal exposure to traffic and household air pollution: a pilot study among street traders in Lagos, Nigeria
Source: J Glob Health. 2026 Apr 17;16:04114. doi: 10.7189/jogh.16.04114 (PMC13086488; doi:10.7189/jogh.16.04114)

Supplement to: Ozoh OB, Amegbor P, Dede S, Adeyeye O, Ekete O, Adetona O, Barrat B. Quantifying personal exposure to traffic and household air pollution: a pilot study among street traders in Lagos, Nigeria. J Glob Health. 2026; 16:04114.

Figure S1. Exposure monitoring bespoke backpack measuring PM_{10}, $PM_{2.5}$, and nitrogen dioxide. Source:

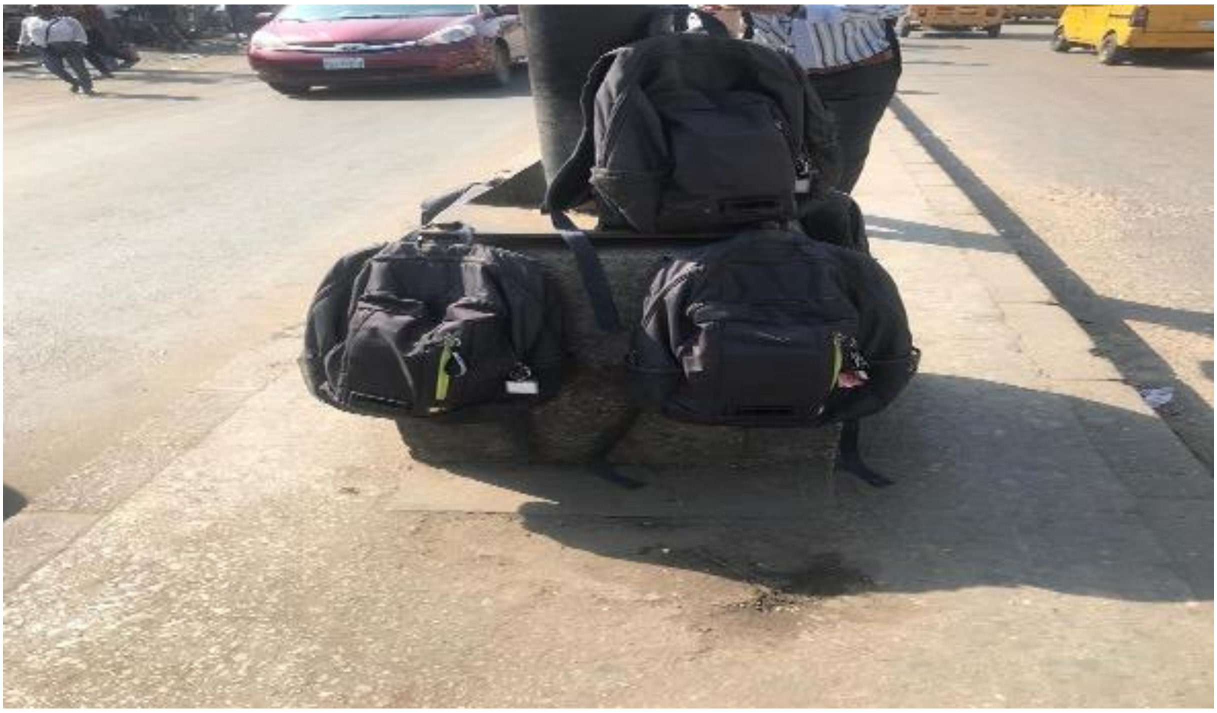

Supplement: Online Supplementary Document [file jogh-16-04114-s001.pdf]
